# Deep Learning-Based Localization of EEG Electrodes Within MRI Acquisitions

**DOI:** 10.3389/fneur.2021.644278

**Published:** 2021-07-08

**Authors:** Caroline Pinte, Mathis Fleury, Pierre Maurel

**Affiliations:** Univ Rennes, Inria, CNRS, Inserm, Empenn ERL U1228, Rennes, France

**Keywords:** EEG, fMRI, electrode detection, electrode labeling, deep learning, U-Net, ICP

## Abstract

The simultaneous acquisition of electroencephalographic (EEG) signals and functional magnetic resonance images (fMRI) aims to measure brain activity with good spatial and temporal resolution. This bimodal neuroimaging can bring complementary and very relevant information in many cases and in particular for epilepsy. Indeed, it has been shown that it can facilitate the localization of epileptic networks. Regarding the EEG, source localization requires the resolution of a complex inverse problem that depends on several parameters, one of the most important of which is the position of the EEG electrodes on the scalp. These positions are often roughly estimated using fiducial points. In simultaneous EEG-fMRI acquisitions, specific MRI sequences can provide valuable spatial information. In this work, we propose a new fully automatic method based on neural networks to segment an ultra-short echo-time MR volume in order to retrieve the coordinates and labels of the EEG electrodes. It consists of two steps: a segmentation of the images by a neural network, followed by the registration of an EEG template on the obtained detections. We trained the neural network using 37 MR volumes and then we tested our method on 23 new volumes. The results show an average detection accuracy of 99.7% with an average position error of 2.24 mm, as well as 100% accuracy in the labeling.

## 1. Introduction

Functional magnetic resonance imaging (fMRI) is a technique that allows to visualize brain activity by detecting hemodynamic variations. It is a non-invasive method that is widely used for the study of brain function [see for example (1)]. Moreover, electroencephalography (EEG) is a technique for measuring the electrical activity of the brain by using electrodes placed on the scalp, which is also a non-invasive method, widely used for the diagnosis of brain disorders and the study of neurophysiological activity (2). These two techniques are complementary and can be very relevant in the study of many neurological disorders. In particular, recent studies have shown the contribution that simultaneous EEG-fMRI can make to the understanding and treatment of epilepsy, for example in identifying epileptogenic networks (3–5). Indeed, fMRI has an excellent spatial resolution, in the order of a millimeter, and a lower temporal resolution, in the order of a second, while EEG has a high temporal resolution (milliseconds), but has a lower spatial resolution (6). In fact, source localization in EEG requires the solving of an inverse problem that is sensitive to several parameters (7), one of the main ones being the forward head model used. Another important parameter for the inverse problem is the 3D position of the electrodes on the scalp (8). Indeed, the accuracy of the estimated coordinates of the EEG electrodes impacts the localization of the EEG sources. Position errors lead to inaccuracies in the estimation of the EEG inverse solution (9). This is an even more important issue in the case of studies involving simultaneous EEG/fMRI acquisitions, where several sessions and thus several EEG cap installations can be required. Furthermore, in order to take full advantage of these mixed acquisitions, the registration between EEG and MRI data must be optimal. It is therefore essential to be able to obtain the EEG electrode positions reliably and accurately.

Several methods have been proposed to address this question (10). To begin with, there are semi-automated methods that require manual measurements (11), which are therefore time-consuming and subject to human error. Then, there are methods that require additional material, such as electromagnetic or ultrasound digitizers (12, 13). Finally, in the context of simultaneous EEG/fMRI acquisitions, there are methods that use MR localization of electrodes. In that case, a measurement system external to the EEG, the MRI, is available, but with the following problem: MRI-compatible EEG systems are designed to be as invisible as possible on most MRI sequences. Therefore, some of these methods require manual measurements (14) as well, and others require special equipment (15, 16). More recent studies have proposed the use of an ultra-short echo-time (UTE) sequence in which the electrodes are more visible (17, 18). This type of recently proposed sequences (19, 20) allows to visualize the tissues with a very short T2 and T2⋆, such as cortical bone, tendons and ligaments, and has the side-effect of enabling imaging MR compatible electrode. The introduction of these new sequences opens the door to new methods, more automatic and more easily usable in the clinical routine. Indeed, no additional equipment is required, and the additional acquisition time is quite short, which does not overburden the corresponding EEG-fMRI studies. In (21), the authors proposed a fully automated method based on a segmentation step followed by a Hough transform in order to select the positions of MR-compatible electrodes in an MRI volume using the UTE sequence. This method does not require any additional hardware and is fully automatic, but can be sensitive to scalp segmentation error. Thus, our aim here is to keep the advantages of this method (i.e., generalization and automation) while simplifying the process, which means minimizing the preliminary steps, and improving performance. In this work, we therefore also use a type of UTE sequence to create an automatic method, but study the contribution of machine learning on the electrode detection task.

Therefore, we propose a new two-fold approach based on a combination of deep learning and template-based registration. In fact, our method starts by training a model to detect the position of the electrodes in an MRI volume. This model is based on the U-Net neural network, a fully convolutional neural network whose architecture allows to obtain accurate segmentations (22). As mentioned above, we use a type of UTE sequence: the PETRA (Pointwise Encoding Time reduction with Radial Acquisition) sequence (23), which is gradually becoming the new standard in applications of UTE sequences. Finally, we use the iterative closest point (ICP) (24) algorithm to take into account the geometrical constraints after the deep learning phase, and to obtain labeling of the electrodes.

## 2. Materials

### 2.1. Simultaneous EEG/fMRI

EEG signals were acquired with an MR-compatible 64-channel cap (Brain Products, Gilching, Germany) of a circumference between 56 and 58 cm, with 64 Ag/AgCl electrodes placed in conformity with the extended international 10–20 EEG system, with one additional ground electrode as AFz. Two 32-channel MR-compatible amplifiers (actiCHamp, Brain Products, Gilching, Germany) were used, and the electrodes were attached to small cups of a diameter of 10 mm and a height of 4 mm, inserted in the cap with gel. A particular attention was given to the reduction of electrode impedance and the positioning of the electrodes according to standard fiducial points.

MRI was performed with a 3T Prisma Siemens scanner running VE11C with a 64-channel head coil (Siemens Healthineers, Erlangen, Germany). PETRA acquisitions were obtained using echo-planar imaging (EPI) with the following parameters: Repetition time (TR1)/(TR2) = 3.61/2,250 ms, Inversion Time (TI1)/(TI2) = 1,300/500 ms, Echo Time (TE) = 0.07 ms, Flip Angle 6°, FOV = 300 × 300 mm^2^, 0.9 × 0.9 × 0.9 mm^3^ voxel size, matrix size = 320 × 105, with 60,000 and 30,000 spokes. The acquisition lasted 6 min for the 60K quality and 3 min for the 30K quality. As a result, PETRA images that we used have a size of 320×320×320 mm and a voxel spacing of 0.9375×0.9375 mm. We also acquired a 1 mm isotropic 3D T1 MPRAGE structural scan.

### 2.2. Subjects

We acquired a set of 60 PETRA volumes that came from 20 different subjects, ranging from 2 to 5 images per subject acquired at different sessions (implying a new positioning of the EEG cap), all varying between two quality levels: 30k and 60k spokes. These volumes were divided into two datasets. The first one was used to train a segmentation model, and the second one was used to test the performance of this model. We decided to separate the data by taking 12 subjects for the training dataset and 8 subjects for the test dataset, resulting in 37 training volumes and 23 test volumes.

## 3. Methods

**Figure 1:**
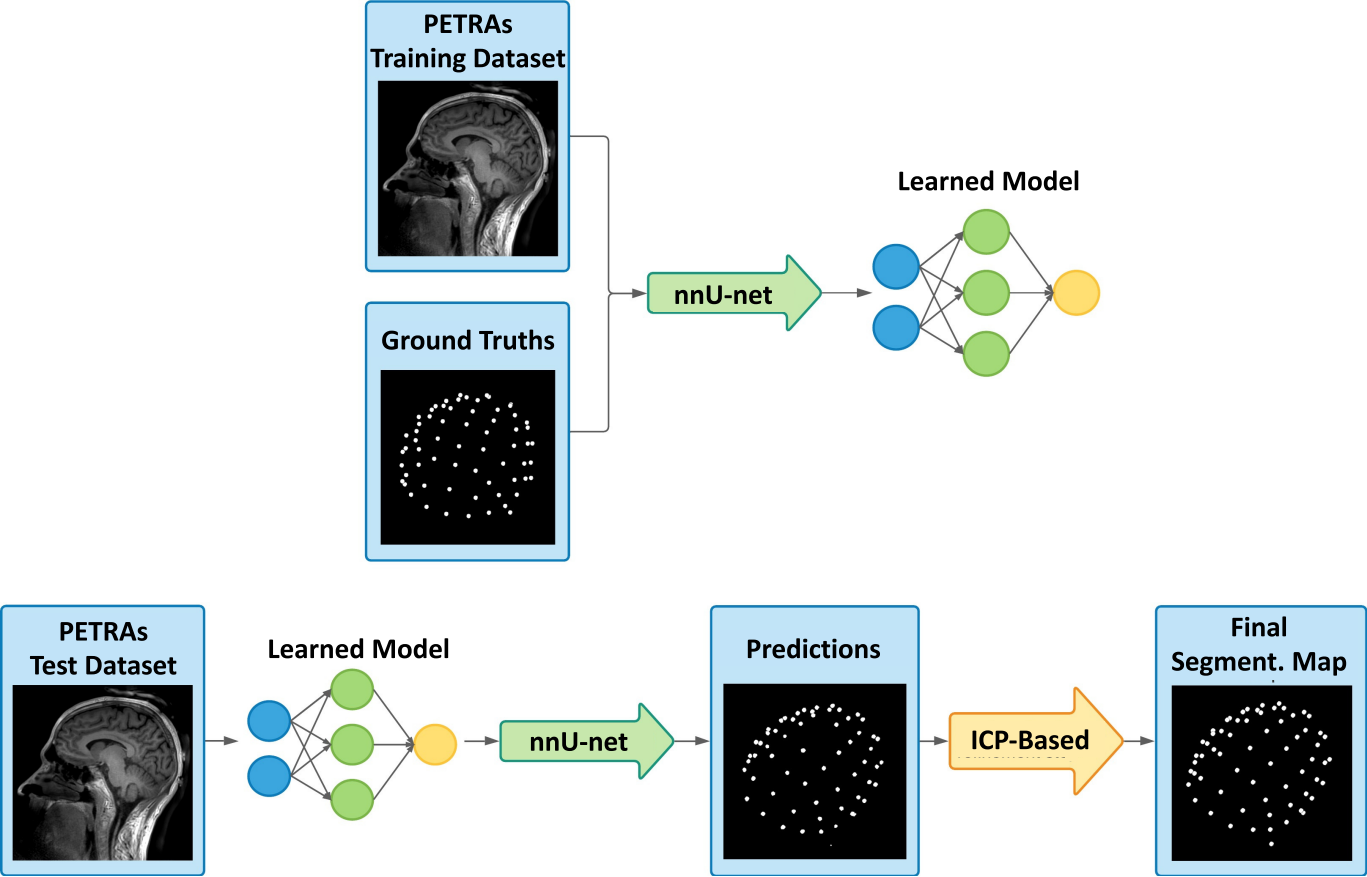
Overview of the presented detection framework, with the learning process **(top)**, and then the deep learning-based prediction and the registration-based refinement step **(bottom)**. From the training dataset and the corresponding labeled ground truths, the deep learning model is trained using the nnU-Net framework. Secondly, our method consists of taking an image never seen by the model and making a predicted segmentation map of the electrodes. Then, template-based adjustments are carried out and the final labeled segmentation map is obtained.

Our two-fold method consists of a first step based on a deep neural network and a second based on a template registration. Figure 1 shows an overview of the method's principles. We will begin by describing how to proceed to train a segmentation model, from data preparation to neural network training by deep learning. Then, we will detail our method for detecting and labeling EEG electrodes on MR images, by explaining how to use the previously trained model as well as the template registration step to obtain the electrode coordinates.

### 3.1. Ground Truth Estimation

To train our model, ground truth segmentation needs to be computed on the PETRA volumes in the training dataset. In our case, ground truths are segmentation maps of the same size and characteristics as the PETRA, with segmented spheres having a different value, also called “label,” for the 65 EEG cap electrodes visible on the scalp and a value of 0 for the background.

To ease the manual creation of these ground truths, a scalp segmentation mask was first estimated. As T1 images have a higher quality than PETRA on the scalp area, this mask is obtained by firstly registering the T1 image on the corresponding PETRA image and then by segmenting the registered T1 image using the FSL library (25). These two inputs allow the use of a Matlab implementation, developed by Butler (26), of a method proposed by de Munck et al. (14) which displays a so-called “pancake” view of the scalp. This colorimetric 2D projection of the scalp region eases the manual selection of the electrode positions. As a result, a 3D labeled segmentation of each PETRA volume was created.

### 3.2. Training Framework

The training dataset thus consists of 37 PETRA images, and their associated ground truth, described above. We use the nnU-Net framework (27). This framework is a tool that can automate the choice of hyperparameters used to train a model from any dataset and for any segmentation task. This is very useful, especially since a large number of variations of neural network architectures have been proposed for segmentation, for example in the biomedical field, and the authors of (27) showed that slight design improvements hardly improve performance, while the choice of hyperparameters seems to be crucial. In fact, this framework with a basic U-Net architecture outperformed most of the specialized deep learning pipelines for 19 international competitions, and 49 segmentation tasks, demonstrating its efficiency but also its adaptability.

Among the different types of neural networks available, we chose the 3D U-Net (28) network whose operations such as convolutions and max pooling are replaced by their 3D counterparts. Once the neural network architecture is chosen, the framework automatically estimates the best training hyperparameters from the dataset provided as input. Here, our model is trained over 1,000 epochs (number of times each training data is considered) and 250 minibatches (number of samples considered before updating internal parameters), with a loss function which is the sum of cross-entropy and Dice loss and with a Stochastic Gradient Descent (SGD) optimizer. The patch used has a size of 128 × 128 × 128 and the default data augmentation scheme provided by nnU-net was used.

### 3.3. Deep Learning-Based Predictions and Template-Based Refinement

Once the model is trained, PETRA images from the test dataset can be provided as input and the model can then perform predictions. The method for making predictions, available in the nnU-net framework, consists of a sliding window approach, using the same patch size that has been used during training, overlapping half of the patch at each step. In order to increase performance, to avoid artifacts, and overall to have a good quality of segmentation, several strategies have been selected: a Gaussian importance weighting is used to reduce edge problems and stitching artifacts, and a so-called “test-time augmentation,” which is data augmentation for test datasets, is used by generating slightly modified images from the tested image and averaging the detections made on them. This data augmentation step is quite time-consuming, so we will compare the results obtained with and without it in the following.

**Figure 2:**
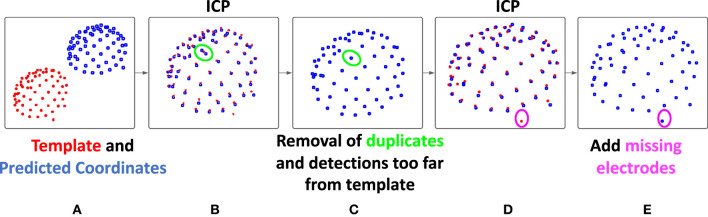
Description of the registration-based refinement step. **(A)** In blue: the prediction points from the deep learning-based step, in red: the template obtained by averaging on the training set, **(B)** a first ICP is performed in order to register the two points cloud, **(C)** for each template point, only the closest detection is kept, **(D)** then, a second ICP is performed and the number of detections is now less than or equal to 65, **(E)** finally, the points in the model not associated with any predictions are added to our final result, which therefore contains exactly 65 detections.

The deep network can take into account spatial information, as well as, naturally, the values present in the image. However, it has more difficulties to incorporate the rather strong geometrical constraint of our problem: the electrodes are all placed on a cap, certainly a little elastic, but the distances between electrodes are, for example, relatively steady. To take into account this geometric constraint, we propose a second step to improve the predictions provided by the neural network. The main objectives of this second step are therefore to force the number of detections to be exactly equal to 65, and to correctly label the electrodes. We start by registering the *n* detections (*n* is not necessarily equal to 65) to an average model of the EEG cap, using the Iterative Closest Point (ICP) algorithm. Figure 2 illustrates the principles of this step.

This template used here is obtained by averaging the coordinates of 12 manually obtained ground truths point clouds from the training set (one per subject, to account for head shape variability). This step then consists of registering these two point clouds (the prediction from the deep learning step and the template) using the ICP algorithm with similarity transformation (rotation, translation, scaling). This registration algorithm (24), between two unpaired point clouds, iterates between two steps. First, each point of the moving set is associated with the nearest point in the fixed set. Then the geometric transformation that minimizes the distance between these pairs of corresponding points is estimated. We then apply this transformation and iterate until convergence.

Then, by comparing the distance between the prediction and the template points, a refinement of the detection is carried out. First, each prediction point is associated with its closest template point, and for each point of the template, only the closest prediction point is kept. As a result of this sub-step, a maximum of 65 predicted positions are conserved. Since only the predictions closest to the model were kept, outliers may have been removed from our initial detections. This is likely to improve the registration, which is why a new ICP is then performed. Finally, using this improved registration, and in the case where less than 65 predictions were kept, the missing positions are added as follows: each template point that is not associated with any prediction positions are added in the final result. Thus, our final result contains exactly 65 detections, each associated with a point of the template, which provides us with a label.

### 3.4. Validation on the Test Dataset

To evaluate the proposed method, and for the test dataset, we compared the detected electrodes to the ground truth coordinates obtained manually. We computed the connected components for the two images and the position of their center. Finally, for each prediction point, its distance to the nearest point of ground truth is computed. This distance is therefore considered a position error. A prediction presenting an error greater than 10 mm, corresponding to the diameter of an electrode cup, is considered as a wrong detection (false positive). Since we systematically consider the nearest ground truth electrode, we do not consider the labeling when estimating the position error. The quality of the final labeling, as well as that of the intermediate labeling, will be evaluated separately. Finally, the number of detections being exactly 65, the number of false positives is automatically equal to the number of missing points (false negatives).

### 3.5. Evaluation of the Robustness of the Method on a Different UTE Sequence

In order to evaluate the robustness of the method, as well as to compare our results with those of (21), we also applied it to images acquired according to a different UTE sequence, the one described in the mentioned article. First, we directly used the model learned from the PETRA images, to study the generalizability of the learned model to another MR sequence. Then, we learned a new model from the different UTE database, containing fewer images, allowing us to investigate the importance of the number of data in the learning set, but also to compare our results to the previously introduced method.

## 4. Results

All the implementations were made on Nvidia Quadro M6000 24GB GPU (which was the most powerful graphics card in 2016 according to NVIDIA Corporation). The training then lasts between 1 and 2 weeks, depending on the number of processes launched on the GPU available. Classically in deep neural network methods, the prediction of one test data is much faster. The presented method predicts a segmentation map from a PETRA image in about 7 min on the above-mentioned GPU.

### 4.1. With Test-Time Augmentation

The results are assessed by measuring the position error as described in the validation section, for all volumes in the test dataset. Table 1 presents the average results for all subjects in the PETRA test dataset. This test set consists of 23 volumes, from 8 different subjects not included in the learning data set, with sampling resolutions of either 30k or 60k spokes, corresponding to a more or less long acquisition time. The average position error is equal to 2.24 mm, to be compared with the diameter of one electrode cup, 10 mm. The number of good (true positive) and wrong (false positive) detections was also assessed, taking that distance of 10 mm as the threshold. As can be seen in the table, after the deep learning step, the number of detections was too high on average, and it was corrected after the registration step, resulting in a better Positive Predictive Value (PPV) defined as the percentage of detections that are true positive relative to the total number of detections.

**Table 1:** Electrodes detection on the test dataset.

	**Deep learning-based detection**	**Final results**
Mean PE (mm)	2.12	2.24
Std PE (mm)	1.50	1.37
Max PE (mm)	8.84	7.99
Mean number of false positives	0.30	0.22
Mean number of true positives	65.0	64.8
PPV (%)	99.5	99.7

*Rows 1,2,3: mean, standard deviation, and maximum values of Position Error (PE). Rows 4,5: mean number of false positives (PE > 10 mm) and true positives (PE ≤ 10 mm). Second column: intermediate results after the deep learning step. Third column: our final results after registration-based refinement step*.

The average total number of detections after the first step is 65.3 (65+0.3) and is therefore higher than the actual number of electrodes (65). This is totally logical since the neural network architecture used does not incorporate any constraint on the number of detections. The output of this first step is a simple volume, where, at each voxel, a label indicates whether it is considered to belong to the background or to a specific electrode. Note that, in this case, two detections associated with the same electrode can count as two good detections, as long as their distance to the said ground truth electrode is less than 10mm. After our registration-based refinement step, the final number of detections is, as expected, exactly equal to 65 (64.8+0.22). Twenty-three volumes were processed, corresponding to a total of 1,495 electrodes, out of which 1,490 were correctly detected and 5 were missed. These missing electrodes often corresponded to those located behind the ears and provoked few outliers in the output. These outliers are reflected in the value of the average maximum error, 8.84 mm. One can note a slight increase of the mean PE after registration. The refinement step indeed usually allows the recovering of some missing electrodes in the intermediate detections provided by the neural network. These new electrodes are therefore provided by the registered model. Although often considered as “true positives” because they are close enough to the ground truth, they are sometimes a little less accurate than the MRI-based detections and cause this relative increase of the mean PE. However, it can be noted that this increase in mean PE comes with a decrease in the standard deviation of position error.

Finally, regarding labeling, 100% of the electrodes were correctly labeled in our final results. As can be seen in the Table 2, this was not the case after the deep learning step. This explains our choice of ICP for the registration step: we cannot always rely on the labeling of intermediate results. Indeed, the number of labeling errors can be as many as 11 in a volume. In fact, these observed errors often correspond to a simple offset in labeling: an electrode is incorrectly labeled and all its neighbors are then likely to be contaminated by this error. We therefore decided to disregard the labeling information provided by the neural network and rely solely on the ICP result for this. It may seem a bit odd to include labels in the ground truth for the training step since we discard the resulting label afterward. Nevertheless, our experiences have interestingly shown that training a neural network with labeled ground truth improves detection results (in terms of position error) compared to a situation where the ground truths are simple binary maps. In particular, in the case where 65 different labels are provided during training, the network is more likely to detect a number close to 65 also during the test phase.

**Table 2:** Electrodes labeling on the test dataset.

**Number of labeling errors among true positives**	**Deep learning- based detection**	**Final results**
Mean	1.87	0
Maximum	11	0

*Number of labeling errors among the true positives, for the intermediate results from deep learning and for our final results*.

### 4.2. Faster Predictions Without Test-Time Augmentation

For each new PETRA image provided, the method presented above allows us to make predictions in about 7 min on our GPU, almost all of this time being used by the first step, based on neural network. As a matter of fact, the ICP-based refinement step runs in few seconds. Therefore, we finally explored the possibility of reducing the computing time required by the neural network to obtain a prediction. To this end, we have removed the test-time augmentation, mentioned in section 3.3. The prediction time of an image was then significantly reduced to about 2 min. Table 3 presents the results of this faster detection pipeline.

**Table 3:** Faster electrode detection on the test dataset.

	**Faster deep learning-based detection**	**Final results**
Mean PE (mm)	6.78	2.23
Std PE (mm)	25.4	1.40
Max PE (mm)	168.7	8.20
Mean number of false positives	2.57	0.13
Mean number of true positives	65.1	64.9
PPV (%)	96.3	99.8

*Rows 1,2,3: mean, standard deviation, and maximum values of Position Error (PE). Rows 4,5: mean number of false positives (PE > 10 mm) and true positives (PE ≤ 10 mm). Second column: intermediate results after the faster deep learning step. Third column: our final results after registration-based refinement step*.

All of the indicators for intermediate results, after the deep learning-based step alone, show that they are clearly worse with this accelerated version: strong increase in Position Error (mean, standard deviation, and maximum values) and increase of the total number of detections. However, the associated detections contain enough valuable information so that the robustness brought by our refinement step allows us to finally obtain results as good as in the first version, as reported in Table 1. Counter-intuitively, some metric values are even slightly better. However, a statistical paired *t*-test showed that none of these changes were significant (*p* > 0.5 for all comparisons).

Finally, and as in the original version, the labeling contained some errors in the intermediate results, but is completely accurate in our final results, even with this faster version, as shown in Table 4. The second step, already important to improve the results in the previous version, turns out to be crucial when we want to accelerate the processing by the neural network, and allows us to obtain similar results.

**Table 4:** Electrodes labeling on the test dataset for the faster version.

**Number of labeling errors among true positives**	**Deep learning- based detection**	**Final results**
Mean	3.2	0
Maximum	13	0

*Number of labeling errors among the true positives, for the intermediate results from deep learning and for our final results*.

### 4.3. Tests on a Different UTE Sequence

In order to evaluate the robustness of our method, we challenged it by testing it on a data set from another MRI sequence, the original UTE one (21). Eleven subjects were included in this new study. A 60k-spokes acquisition was done for all subjects and a 30k-spokes image was acquired for seven of them.

First, the previous model, learned using the PETRA images, was used to detect the electrode positions on these 18 new images, acquired with a different UTE sequence. Results are shown in Table 5. As expected, the detections estimated by the neural network were not as good as in the previous case. Indeed, the average number of electrodes provided was lower than 57. However, and very interestingly, these electrodes were mostly true detections. For this reason, and as can be seen in the table, the ICP-based registration step was able to retrieve almost all missing electrodes, leading once again to excellent performance results. Our registration-based refinement step thus brings robustness to the method, allowing to limits the risk of overfitting, and improving its generalizability.

**Table 5:** Electrodes detection on the UTE dataset, using the previous model, learned using the PETRA images.

	**Deep learning-based detection**	**Final results**
Mean PE (mm)	1.81	2.47
Std PE (mm)	1.67	1.64
Max PE (mm)	11.06	9.36
Mean number of false positives	0.33	0.72
Mean number of true positives	56.4	64.22
PPV (%)	99.4	98.89

*Rows 1,2,3: mean, standard deviation, and maximum values of Position Error (PE). Rows 4,5: mean number of false positives (PE > 10 mm) and true positives (PE ≤ 10 mm). Second column: intermediate results after the deep learning step. Third column: our final results after registration-based refinement step*.

Finally, in order to compare our results to (21), we learned a new neural network, using only this different UTE sequence, applied the refinement step, and evaluated the resulting performance. From the previously described UTE dataset, we built two groups: 9 MRI volumes in the training set and 9 volumes in the test set, again ensuring that no subjects were present in both sets. Table 6 shows the corresponding results. Training the model using the same type of images as in the tests slightly improves the quality of the detections, compared to when training the model on PETRA images. Moreover, and despite this smaller group size (compared to the PETRA study), our results are now better than those reported in (21). For example, the mean PPV is now 99.3% and was between 88 and 94% for 30k and 60k spokes images, respectively.

**Table 6:** Electrodes detection on the UTE dataset, using a new model, learned using images acquired with the same UTE sequence.

	**Deep learning-based detection**	**Final results**
Mean PE (mm)	1.70	2.42
Std PE (mm)	1.24	1.29
Max PE (mm)	8.02	8.19
Mean number of false positives	0.56	0.44
Mean number of true positives	60.0	64.6
PPV (%)	99.1	99.3

*Rows 1,2,3: mean, standard deviation, and maximum values of Position Error (PE). Rows 4,5: mean number of false positives (PE > 10 mm) and true positives (PE ≤ 10 mm). Second column: intermediate results after the deep learning step. Third column: our final results after registration-based refinement step*.

For both of these cases, all the detected electrodes were once again well-labeled: there was no mislabeling among the true positives.

## 5. Discussion

We have introduced a new fully automatic method for the detection of EEG electrodes in an MRI volume during simultaneous EEG-MRI acquisition. This technique is easy to set up and use, and gives accurate and reliable results. Indeed, after the model has been learned once and for all, the method requires nothing more than acquiring a PETRA volume, after the installation of the EEG headset. No additional equipment is required, and the PETRA volume can be acquired in a few minutes. The computation time is, for the most part, used by the deep learning-based prediction. This can be accelerated up to 2 min and is the most important part of the proposed method. Nevertheless, as the results showed, the second registration-based step allows both to improve the final results and to make them more robust to possible outliers.

It is well-known that deep learning models are highly dependent on the quality and representativeness of the data in the learning set. Our first investigations in this direction, using a different UTE sequence, seem to indicate that the method can be generalized to other types of images, even keeping the model learned on the initial data, thanks to the robustness brought by the registration step. Another interesting question is the behavior of the method when the number of electrodes is not the same between the learning and testing phases. One can hope that the robustness brought by the second ICP-based step can provide a good detection, if the same sequence and the same type of electrodes are used, but this needs to be verified with a future investigation. Finally, this method has been tested on one type of EEG cap (Brain Products), but is valid for any detection problem of elements on the scalp. It will therefore also be interesting to test it on other EEG headsets, but also on other systems, for example, the near-infrared spectroscopy (NIRS) modality, which consists of a system of optodes placed on the scalp.

Finally, it should also be noted that our second study, on the original UTE sequence, had a smaller sample size, probably more consistent with a typical simultaneous EEG-MRI study (11 subjects were involved, corresponding to 18 volumes, and only 9 of these were used in the training phase). Despite the smaller amount of data, the results (Table 6) were only slightly less good than those obtained with a larger sample (Table 1).

## 6. Conclusion

We presented a new method for the detection and labeling of EEG electrodes in an MR volume acquired using PETRA sequence. The first step is to train a model from a set of training data and associated manual ground truths, then use this model to obtain a segmentation map, and finally to apply a step using the ICP registration algorithm to improve the detections and their labeling. This fully automatic method is easy to implement, requires very few steps, and gives excellent results. For all these reasons, we strongly believe that it can be very useful for all protocols with simultaneous EEG-fMRI acquisitions. In particular, when an EEG source localization is planned later, as is often the case when studying epilepsy, accurate information on the position of the electrodes is a definite advantage.

## Data Availability Statement

The datasets presented in this article are not readily available because of patient data. Requests to access the datasets should be directed to the corresponding author.

## Ethics Statement

The studies involving human participants were reviewed and approved by Comite de Protection des Personnes Ouest V Rennes. The patients/participants provided their written informed consent to participate in this study.

## Author Contributions

CP, MF, and PM: conception of the method and manuscript writing. CP: implementation. All authors contributed to the article and approved the submitted version.

## Conflict of Interest

The authors declare that the research was conducted in the absence of any commercial or financial relationships that could be construed as a potential conflict of interest.
